# GPR137 inactivates Hippo signaling to promote gastric cancer cell malignancy

**DOI:** 10.1186/s13062-023-00449-8

**Published:** 2024-01-02

**Authors:** Lin Li, Jinlong Tang, Bin Cao, Qiang Xu, Shouying Xu, Chao Lin, Chao Tang

**Affiliations:** 1 National Clinical Research Center for Child Health, The Children’s Hospital, Zhejiang University School of Medicine, No. 3333, Binsheng Road, Hangzhou, 310052 People’s Republic of China; 2 Department of Urology, Third Affiliated Hospital of the Second Military Medical University, Shanghai, 201805 People’s Republic of China; 3 Department of Pathology, The Second Affiliated Hospital, Zhejiang University School of Medicine, Hangzhou, 310005 People’s Republic of China; 4 Zhejiang Provincial Center for Disease Control and Prevention, Hangzhou, People’s Republic of China; 5 Department of Neurosurgery, The Children’s Hospital, Zhejiang University School of Medicine, Hangzhou, 310052 People’s Republic of China

**Keywords:** Gastric cancer, GPR137, Hippo, MST

## Abstract

As the fifth most common cancer in the world, gastric cancer (GC) ranks as the third major cause of cancer-related death globally. Although surgical resection and chemotherapy still remains the mainstay of potentially curative treatment for GC, chemotherapy resistance and adverse side effects limit their clinical applications. Thus, further investigation of the mechanisms of carcinogenesis in GC and discovery of novel biomarkers is of great concern. We herein report that the elevated expression of GPR137 is correlated with GC. Overexpression of GPR137 potentiates human gastric cancer AGS cell malignancy, including proliferation, migration, invasion, colony formation and xenograft growth in nude mice in vivo, whereas knockout of *GPR137* by CRISPR/Cas9 gene editing exerts the opposite effects. Mechanistically, GPR137 could bind to MST, the upstream kinases in Hippo pathway, which disrupts the association of MST with LATS, subsequently activating the transcriptional co-activators, YAP and TAZ, and thereby triggering the target transcription and the alterations in GC cell biological actions consequently. Therefore, our findings may provide with the evidence of developing a potentially novel treatment method with specific target for GC.

## Introduction

As the fifth most common cancer around the world, gastric cancer (GC) ranks as the third major cause of cancer-related death globally, with a particularly high incidence in Asian countries including China and Japan [1, 2]. Despite the declining incidence of GC, there are still over 1 million cases newly diagnosed and 850,000 deaths globally each year [3]. Mainly due to the lack of specific symptoms and appropriate diagnostic markers in early stage GC, patients with GC are always diagnosed at an advanced stage. In western countries, the 5-year survival of GC patients ranges from 5 to 20%, whereas in Asia such as Japan, the survival rate is about 50% due to early diagnosis [4]. Although clinical treatments such as molecular-targeted therapy have emerged, surgical resection and adjuvant chemotherapy still remains the mainstay of potentially curative treatment for GC. Thus, further investigation of the mechanism of carcinogenesis in GC and discovery of novel biomarkers is of great concern for early diagnosis and for improving the prognosis and survival of patients with advanced GC.

G protein-coupled receptor 137 (GPR137, also known as C11orf4, GPR137A or TM7SF1L1) belongs to the member of cell surface mediators of signal transduction [5, 6]. *GPR137* gene located in 11cen-q13.1 was originally discovered using custom search of the GenBank genomic databases based on known G protein-coupled receptor (GPCR) coding sequences [6]. The *GPR137* gene encodes a peptide with four trans-membrane domains in the cytoplasm, and GPR137 protein contains signal peptides at both the N-termini and the C-termini. Although transcription of *GPR137* was first detected in the human hippocampus, GPR137 expression was later also found to be ubiquitously and abundantly distributed in other organs and tissues, such as the endocrine, reproductive, and pulmonary systems [7], suggesting that GPR137 may be involved in a variety of physiological activities.

As one of the novel human GPCRs that play a vital role in cell signaling transduction, recently studies have identified that GPR137 expression is closely associated with cancer and the aberrantly up-regulated expression of GPR137 is correlated with carcinogenesis in kinds of cancers, such as prostate cancer [6], ovarian cancer [8], pancreatic cancer [9] as well as osteosarcoma [10], indicating the potential role of GPR137 as a novel tumor biomarker. However, the function of GPR137 in GC remains largely elusive.

In our study presented here, we first exam the expression of GPR137 in clinical samples from GC patients, and find that the expression of GPR137 is significantly up-regulated in GC tissues, compared with the adjacent normal tissues. Consistently, overexpression of GPR137 potentiates human GC AGS cell proliferation, migration, invasion, colony formation as well as AGS cell-derived xenograft growth in nude mice, whereas knockout of *GPR137* by CRISPR/Cas9 gene editing method exerts the opposite effects on AGS cells. Mechanistically, we elucidate that the GPR137-medicated GC cell malignancy is through its regulation of Hippo signaling, particularly by its binding to MST kinases, which dampens the MST-LATS protein–protein complex formation and subsequently triggers the transactivation of YAP/TAZ-mediated downstream target genes, consequently resulting in the enhanced cancer cell malignancy.

## Materials and methods

### Cell lines and cell culture

Human normal gastric epithelial GES-1 cells, human gastric cancer AGS cells and human embryonic kidney 293T (HEK293T) cells were obtained from ATCC (Manassas, VA). Human gastric cancer SGC-7901 cells were purchased from Beyotime Institute of Biotechnology (Shanghai, China). AGS cells were cultured in RPMI 1640 medium (Hyclone, USA) supplemented with 10% (v/v) fetal bovine serum (FBS, Life Technologies, Inc., Grand Island, NY) at 37 °C with 5% CO_2_ as described previously [11]. GES-1, SGC-7901 and HEK293T cells were maintained in high glucose DMEM (Life Technologies, Inc., Grand Island, NY) supplemented with 10% (v/v) FBS at 37 °C with 5% CO_2_ as described previously [11–13].

### CRISPR/Cas9 to GPR137 construction and transfection

Expression vector of sgRNA for human *GPR137*, human *MST1* or human *MST2* was designed as pX330-based plasmids. All specific target sgRNA sequences were amplified and cloned, and verified by DNA sequencing. After the transient transfection of pX330-sg*GPR137* plasmid, or pX330-sg*MST1* and pX330-sg*MST2* plasmids together with a puromycin-resistant plasmid into cells by using Lipofectamine reagent (Invitrogen), puromycin (2 μg/ml) (Invitrogen) treatment for 7 d was employed for selection and then cells were expanded in the regular culture medium.

### Oligonucleotides, plasmids, viruses and infections

The primers for quantitative RT-PCR (qRT-PCR) were as follows:

human *GPR137*-F-5’-ACCTGGGGAACAAAGGCTAC-3’;

human *GPR137*-R-5’-TAGGACCGAGAGGCAAAGAC-3’;

human *YAP*-F-5’-GGATTTCTGCCTTCCCTGAA-3’;

human *YAP*-R-5’-GATAGCAGGGCGTGAGGAAC-3’;

human *TAZ*-F-5’-TCATCACCGTGTCCAATC-3’;

human *TAZ*-R-5’-CTGAAGAAGTGGGAGTGTAG-3’;

human *CTGF*-F-5’-CAACTGCCTGGTCCAGACC-3’;

human *CTGF*-R-5’-CACTCTCTGGCTTCATGCC-3’;

human *CYR61*-F-5’-CTTACGCTGGATGTTTGAGTGT-3’;

human *CYR61*-R-5’-AGACTGGATCATCATGACGTTCT-3’;

human *GAPDH*-F-5’-CCTCAACTACATGGTTTACATGTTCC-3’;

human *GAPDH*-R-5’-GAAGATGGTGATGGGATTTCCATTG-3’.

The GPR137 lentiviral expression vector, pCDH-CMV-MCS-EF1-GPR137-copGFP, was constructed by Mr. Qiang Xu, and the pCDH-CMV-MCS-EF1-copGFP empty vector was used as a control. Lentiviruses expressing GPR137 were generated as described previously [14], and the lentiviruses-containing supernatants with the titers greater than 1 × 10^6^ cfu/ml was applied for infection of human AGS cells in the presence of 8 μg/ml polybrene (Sigma, St. Louis, MO, USA).

### Antibodies and chemicals

Phospho-MST1 (pMST1-Thr183, bs-4635R), MST1 (bs-3504R), MST2 (bs-4663R), phospho-LATS1 (pLATS1-Thr1079, bs-3245R), GPR137 (bs-16270R), PCNA (bs-2007R) antibodies were from Bioss (Beijing, China). Phospho-LATS2 (pLATS2-Ser872, AP0904) antibody was from ABclonal, (Wuhan, China), Flag (M185-3) antibody was from MBL (Beijing, China), and antibodies for CTGF (sc-365970), CYR61 (sc-374129), GAPDH (sc-32233) and normal mouse IgG (sc-2025) were from Santa Cruz Biotechnology (Santa Cruz, CA, USA). LATS1 (#3477), LATS2 (#5888), phospho-YAP (pYAP-Ser127, #13008), YAP (#14074), TAZ (#72804) antibodies, and normal rabbit IgG (#2729) were purchased from Cell Signaling Technology (Danvers, MA, USA) and phospho-TAZ (pTAZ-Ser-89, PA5-105066) antibody was purchased from Thermo Fisher Scientific. HA-tag antibody (AF2305) was from Beyotime Institute of Biotechnology (Shanghai, China). XMU-MP-1 was from Selleck (Shanghai, China).

### RNA isolation, reverse transcription and quantitative RT-PCR (qRT-PCR)

Total RNA was isolated from AGS cells and SGC-7901 cells by using a Trizol reagent (Takara Biotechnology Co., Ltd., Dalian, China) as per the manufacturer’s instructions. 5 μg total RNA in a volume of 20 μl was reversely transcribed by using a SuperScript III reagent (Life Technologies) and the oligo-(deoxythymidine) primer with incubation at 42 °C for 1 h. After the termination of cDNA synthesis, each reaction mixture was diluted with 80 μl Tris–EDTA buffer. qRT-PCR was subsequently performed to measure the expression of *GPR137*, *CTGF* and *CYR61*. The relative amounts of the mRNA levels were normalized to the *GAPDH* levels, respectively, and the relative difference in mRNA levels was calculated by 2^−△△ct^ method as described previously [15, 16].

### Transient transfection and TEAD-luciferase reporter assay

Transient transfections were conducted by using Lipofectamine 2000 reagent (Invitrogen, Thermo Fisher Scientific) as per the manufacturer’s instructions. TEAD-luciferase reporter activities were measured by using the dual luciferase reporter assay (Promega) as per the manufacturer’s protocol as described previously [11]. A pRL/TK-luciferase reporter plasmid was used as a second reporter for normalization of the results. The data were obtained by analyzing triplicated samples.

### Nucleo-cytoplasmic separation assay, western blot and co-immunoprecipitation

For nucleo-cytoplasmic separation assay, the nuclear and cytosolic fractions of AGS cells were prepared as described previously [14]. Western blots were performed using standard protocols. Briefly, total protein extracts were prepared, and protein concentrations were determined by using a standard Bradford assay. 50 μg of total protein was subjected to SDS-PAGE followed by a transfer onto PVDF membranes (Millipore, Bedford, MA). Membranes were incubated with primary antibodies at 4 °C overnight followed by incubation in secondary antibodies at room temperature for 2 h. The intensity of protein bands was quantified using NIH ImageJ software (ImageJ, http://rsb.info.nih.gov/ij/). Co-immunoprecipitation was performed as described previously [15]. Briefly, AGS cells were harvested and lysed in lysis buffer containing 100 mM NaCl, 50 mM Tris–HCl (pH 8.0), 5 mM EDTA, 1% Brij35, 2 mM Na_3_VO_4_, 10 mM NaF, 2 mM β-glycerophosphate and 2 mM PMSF, and were incubated with respective antibodies and protein A/G plus agarose (sc-2003, Santa Cruz). The beads were then washed five times with the lysis buffer, and the immune complex was eluted with western blot sample buffer. Lysates and immunoprecipitates were subjected to western blot.

### Immunofluorescence staining

Immunofluorescence staining was performed on chamber slides (Nalge Nunc International, Naperville, IL) as described previously [17]. Plasmids-transfected and selected AGS cells were fixed in ice-cold methanol and were then permeabilized with 0.1% TritonX-100 in PBS (PBST). After blocking with BSA solution, AGS cells were incubated with HA antibody and subsequently with fluorescent secondary antibody. Nuclei were counterstained with 4',6-diamidino-2-phenylindole (DAPI). Slides were analyzed by a laser scanning microscope (Zeiss, Germany). GPR137 expression was scored as previously described [18] based on the proportion of cells showing GPR137 immunostaining across 3 non-adjacent fields in each sample with the following criteria: 0 (no cell positive); 1 (< 50% cells weakly positive); 2 (< 50% cells intensely staining); 3 (≥ 50% cells weakly positive); 4 (≥ 50% cells intensely staining).

### Cell proliferation by cell counting kit-8 (CCK-8) assays

CCK-8 assay was performed according to the manufacturer’s protocol (Yeasen, Shanghai, China) as described previously [19]. After treatment, AGS cells were incubated with 10 μl CCK-8 reagent for 2 h at 37 °C and the absorbance was measured at a wavelength of 450 nm with a micro-plate reader.

### Wound healing assay

Wound healing assay was performed as previously described [20]. Briefly, AGS cells (4 × 10^5^ cells) were cultured in six-well plate and were transfected with indicated plasmids. 24 h after transfection, cells were subjected to serum starvation for 12 h. After rinsed with medium to remove unattached cells, the confluent layer of cells was scratched with a sterile tip to create an artificial wound. Cell migration to the wounded gap was then monitored by microscopy after 24 h and the distance between the edges of the wound was analyzed using ImageJ software.

### Matrigel invasion assay

Invasion of cells was measured in Matrigel (BD Biosciences, NJ)-coated Transwell inserts (6.5 μm, Costar, Cambridge) containing polycarbonate filters with 8 μm pores as detailed previously [16] Briefly, the mixture of Matrigel and medium at the proportion of 1:2 at 50 µl was enclosed by each transwell membrane. After infection for 48 h, AGS cells (2 × 10^5^) in 200 μl of serum-free medium were plated in the upper chamber, whereas 600 μl of medium with 10% FBS was added to the lower well. After incubation for 24 h, non-invading cells that remained on the upper surface of the filter were removed, and the cells that had passed through the filter and attached to the bottom of the membrane were fixed in methanol and stained with 0.2% crystal violet. Numbers of the invasive cells in six randomly selected fields from triplicate chambers were counted in each experiment. The migrated cells were counted under a light microscope.

### Colony formation assay

Colony formation assay was performed as previously described [11]. Briefly, AGS cells were transfected with indicated vectors. At 24 h after transfection, the cells were seeded in 6-well plates at a density of 1000 cells/well in 2 ml culture medium. After culturing for two weeks, colonies were stained with 0.5% crystal violet in 2% ethanol, and colonies were counted, photographed and statistically analyzed.

### AGS-xenografts and treatments

Female Balb/c-nu/nu mice (6-week-old) were rested for a week, and tumor-bearing mice model was established by subcutaneous inoculation with xenografts of human AGS cells (5 × 10^6^ cells/site) infected with control lentiviruses or GPR137-expressing lentiviruses into the left armpit. At day 14 after inoculation, the mice were sacrificed, and the tumors were harvested for further analysis. All animal care and handling procedures were approved by the Institutional Animal Care and Use Committee of Zhejiang University.

### Immunohistochemistry staining

Immunohistochemistry staining was performed by using the Histostain-Plus Kit (Kangwei Reagents, Beijing, China) according to the manufacturer’s instructions. Briefly, paraffin-embedded tumor Sects. (4 μm) were deparaffinized and rehydrated in xylene and a graded series of ethanol. After antigen retrieval in 10 mM sodium citrate and 10 mM citric acid, tissue sections were then incubated with 3% H_2_O_2_ in methanol to quench endogenous peroxidase followed by sequential incubation including with normal serum for 30 min, with primary antibodies against GPR137 or TAZ at 4 °C overnight, and with HRP-labeled secondary antibody (Life Technologies) for 30 min. The diaminobenzidine (DAB) solution was used for development of color, and the sections were counterstained with hematoxylin.

### Obtainment of human tissue samples

Total of 10 pairs of GC tissues and adjacent non-tumor tissues were collected from patients undergoing surgery at the Second Affiliated Hospital of Zhejiang University School of Medicine from 2021 to 2022. Patients didn’t receive chemotherapy or radiotherapy before operation. Pathologists identified all tumor tissues and paired adjacent non-tumor tissues. This study was conducted under a protocol approved by the Second Affiliated Hospital, Zhejiang University School of Medicine institutional review board, and informed consent was obtained from each patient.

### RNA-seq analysis

A minimum of 3 μg of total RNA from AGS cells was oligo (dT) selected using the Dynabeads mRNA purification kit (Invitrogen). Then, the mRNA isolated from total RNA was fragmented into short fragments with a fragmentation buffer (Ambion), and double-stranded cDNA was synthesized with these short fragments as templates. Next, the cDNA was end-repaired, ligated to Illumina adapters, size selected on agarose gel (approximately 250 bp) and PCR amplified. The cDNA library was subsequently sequenced on an Illumina HiSeq 2000 sequencing platform (Berry Genomics). The gene expression levels for each transcript were estimated as the number of reads per kilo-base of exon model per million mapped reads (RPKM) using only uniquely mapped reads in exonic regions. A gene is considered significantly differentially expressed if its expression differs between samples from the two groups, control group and GPR137-overexpression group, with the log Fold Change > 1 or <  − 1 and the *p* value < 0.05 as calculated by Cufflinks.

### Bioinformatics analysis

The expression of GPR137 [log2(TPM + 1)] in stomach adenocarcinoma (STAD) tissues and normal stomach tissues was assessed in accordance with GEPIA database (http://gepia.cancer-pku.cn). In addition, GEPIA online tool was used to produce the correlations between STAD patient GPR137 and CTGF expression as well as GPR137 and CYR61 expression with auto best cutoff.

### Statistics analyses

Data were expressed as mean ± S.D. and were analyzed by one-way ANOVA and Tukey–Kramer multiple comparison test (SPSS 13.0J software; SPSS, Inc., Chicago, IL). Statistical significance was assessed at *p* < 0.05 and *p* < 0.01. Experiments were independently triplicated and representative experiments are shown.

## Results

### GPR137 expression is up-regulated in GC

**Fig. 1:**
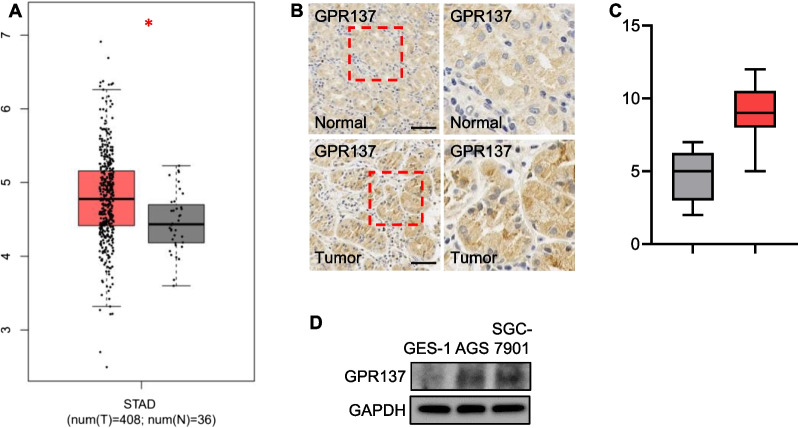
Up-regulated GPR137 expression is associated with GC. **A** Expression of GPR137 in STAD tissues and control groups was revealed by GEPIA database. *p < 0.05; error bar, SD. **B** Immunohistochemistry staining for GPR137 by using paraffin-embedded sections of cancer tissues and adjacent normal gastric epithelial tissues from patients with GC. N = 10, bar = 40 μm. **C** IHC score for GPR137. N = 10. **D** The protein expression levels of GPR137 in human normal gastric epithelial GES-1 cells, human gastric cancer AGS cells and SGC-7901 cells. One representative of 3 independent blots is shown

To first examine the expression of GPR137 in tumor tissue from GC patients, we utilized the database online. According to Gene Expression Profiling Interactive Analysis (GEPIA) database, GPR137 expression was significantly up-regulated in stomach adenocarcinoma (STAD, accounting for around 90–95% of all GC [21]) tissue samples (T, Tumor) in comparison with control group (N, Normal) (Fig. 1A), and the elevated expression of GPR137 was further validated by immunohistochemistry staining, showing the increased GPR137 expression in STAD samples than matched controls (adjacent normal tissues, Fig. 1B,C). Consistently, the protein expression of GPR137 was obviously higher in human gastric cancer AGS cells and SGC-7901 cells than that in human normal gastric epithelial GES-1 cells (Fig. 1D). The above data suggest that the aberrantly elevated expression of GPR137 is correlated with GC.

### GPR137 potentiates AGS cell malignancy

**Fig. 2:**
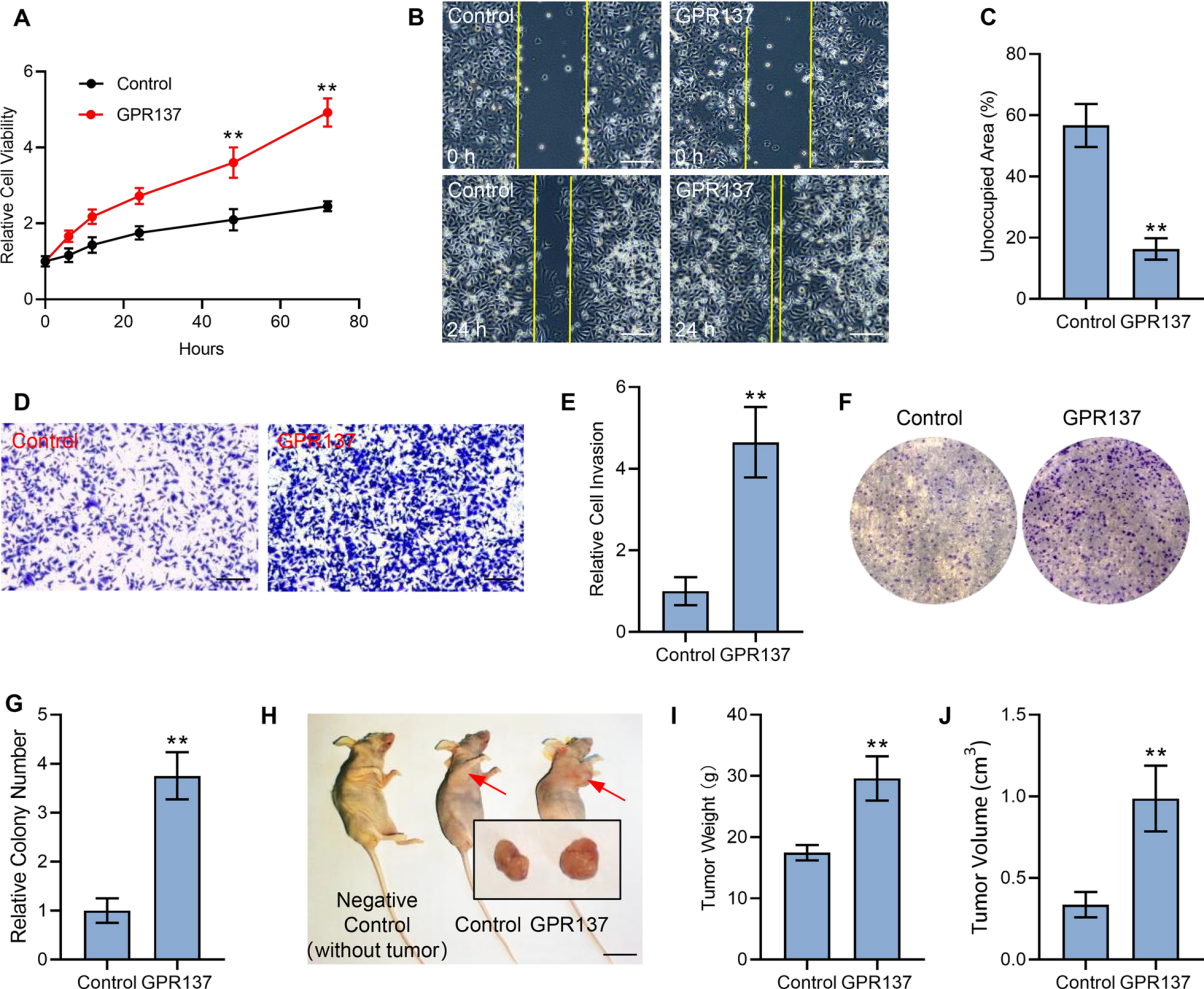
GPR137 promotes AGS cell malignancy. **A** CCK-8 assays of AGS cells transfected with GPR137 or an empty vector (control) and cultured for different periods. **p < 0.01; error bar, SD. N = 3. **B** Wound healing assays of AGS cells transfected with GPR137 or an empty vector (control) at 24 h. Bar = 100 μm. N = 3. **C** Statistical analysis of unoccupied area in (**B**). **p < 0.01; error bar, SD. **D** Matrigel invasion assays of AGS cells transfected with GPR137 or an empty vector (control) for 24 h. Bar = 100 μm. N = 3. **E** Quantitative analysis of (**D**). **p < 0.01; error bar, SD. **F** Colony formation assays of AGS cells transfected with GPR137 or an empty vector (control). N = 3. **G** Quantitative analysis of (**F**). **p < 0.01; error bar, SD. **H** Nude mice with xenografts derived from AGS cells infected with GPR137-expressing lentiviruses (GPR137) or control viruses (Control). **I**, **J** Tumor weight (**I**) and tumor volume (**J**) in (**H**). N = 6; **p < 0.01; error bar, SD

To assess the potential role of GPR137 in GC cells, we transfected a GPR137-expressing plasmid into AGS cells, a common used human cell line for GC investigation [22]. Our data showed that overexpression of GPR137 markedly enhanced AGS cell proliferation in a time-dependent manner, particularly at 48 h and 72 h (Fig. 2A). In addition, overexpression of GPR137 significantly accelerated gap closure in AGS cells (Fig. 2B,C), which was examined by wound healing assay, and, the ectopic expression of GPR137 apparently promoted AGS cell invasion (Fig. 2D,E) and stimulated AGS cell colony formation (Fig. 2F,G), which was determined by matrigel-transwell assay and colony formation assay, respectively. Consistently, compared with the control AGS cells, the GPR-137-expressing lentiviruses-infected AGS cells resulted in the xenografts with the significantly increased tumor weight and tumor volume in nude mice (Fig. 2H–J), suggesting that GPR137 regulates AGS cell malignancy both in vitro and in vivo. Based on these results, we concluded that GPR137 plays an important role in GC.

### Transcriptome analysis of GPR137-regulated genes

**Fig. 3:**
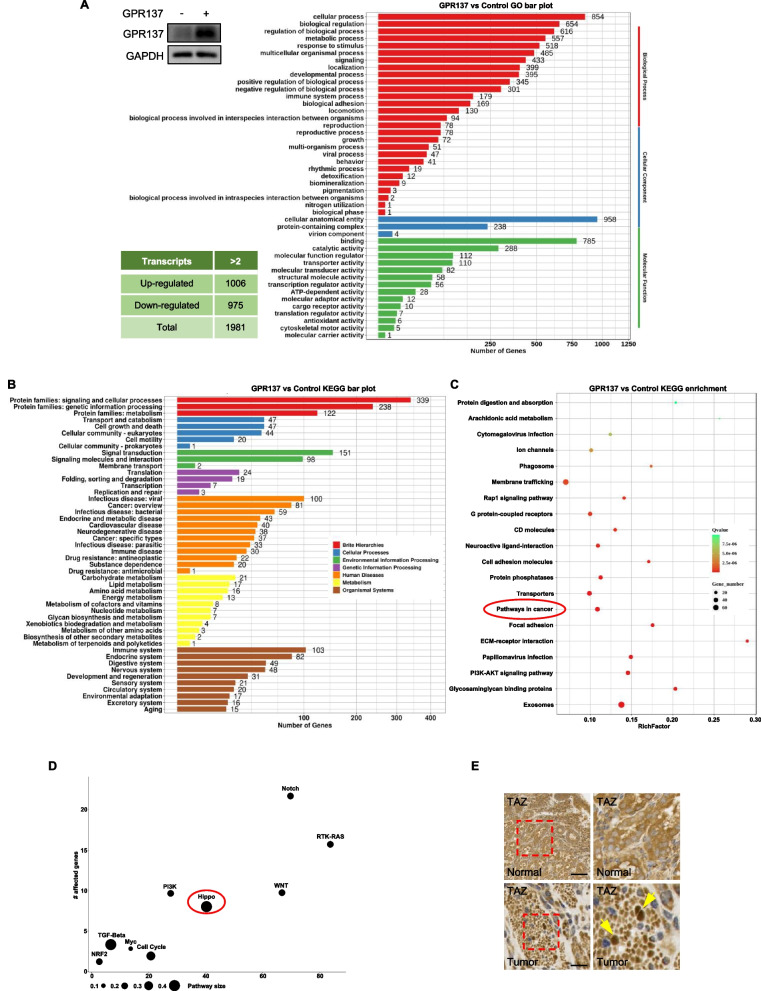
Transcriptome analysis of GPR137-regulated genes. **A** Protein levels of GPR137 in AGS cells transfected with GPR137 (GPR137, +) or an empty vector (GPR137, -) for 24 h were detected by western blot. The number of genes whose expression was altered by GPR137 is summarized in the table, and GO analysis was performed. One representative of 3 independent blots is shown. **B** KEGG was conducted to compare the gene expression from GPR137 group and the control. **C** Pathway enrichment analysis of (**B**). **D** Top cancer-related pathways were shown. **E** Immunohistochemistry staining for TAZ by using paraffin-embedded sections of cancer tissues and adjacent normal gastric epithelial tissues from patients with GC. N = 10, bar = 40 μm

To gain insights into the molecular mechanisms underlying the role of GPR137 in GC, RNA-seq was performed in AGS cells transfected with a GPR137-expressing vector or a control empty vector, after the up-regulated GPR137 protein levels were determined by western blot (Fig. 3A). A total of 1981 transcripts (log Fold Change > 1 or <  − 1) were significantly changed upon GPR137-overexpression, among which 1006 were up-regulated and 975 were down-regulated (Fig. 3A), respectively, indicating that variation of levels of GPR137 expression profoundly altered the transcriptome. Results of Gene Ontology (GO) and Kyoto Encyclopedia of Genes and Genomes (KEGG) analysis suggested that the ectopic expression of GPR137 affected many important processes in cell biology, such as cell signaling transduction, cell growth and cell motility (Fig. 3A,B). As expected, GPR137 was found to be associated with diseases, including cancer (Fig. 3B, Cancer: overview; Cancer: specific types). In addition, the enrichment analysis of the top 20 pathways from KEGG also exhibited that GPR137 was correlated with multiple pathways in cancer (Fig. 3C), including Notch, RTK-RAS, WNT, Hippo, PI3K, Myc, TGF-Beta, NRF2 and cell cycle signaling, among which Hippo signaling revealed the largest pathway size with more affected genes (Fig. 3D). Consistently, immunohistochemistry staining data showed that the localization of TAZ, a key transcriptional co-activator in Hippo signaling, was mainly distributed in nucleus in GC tissues while in cytoplasm in normal gastric epithelial tissues (Fig. 3E), which was in agreement with a previous report [23]. Thus, GPR137 may affect Hippo signaling in GC.

### GPR137 inhibits Hippo signaling activity

**Fig. 4:**
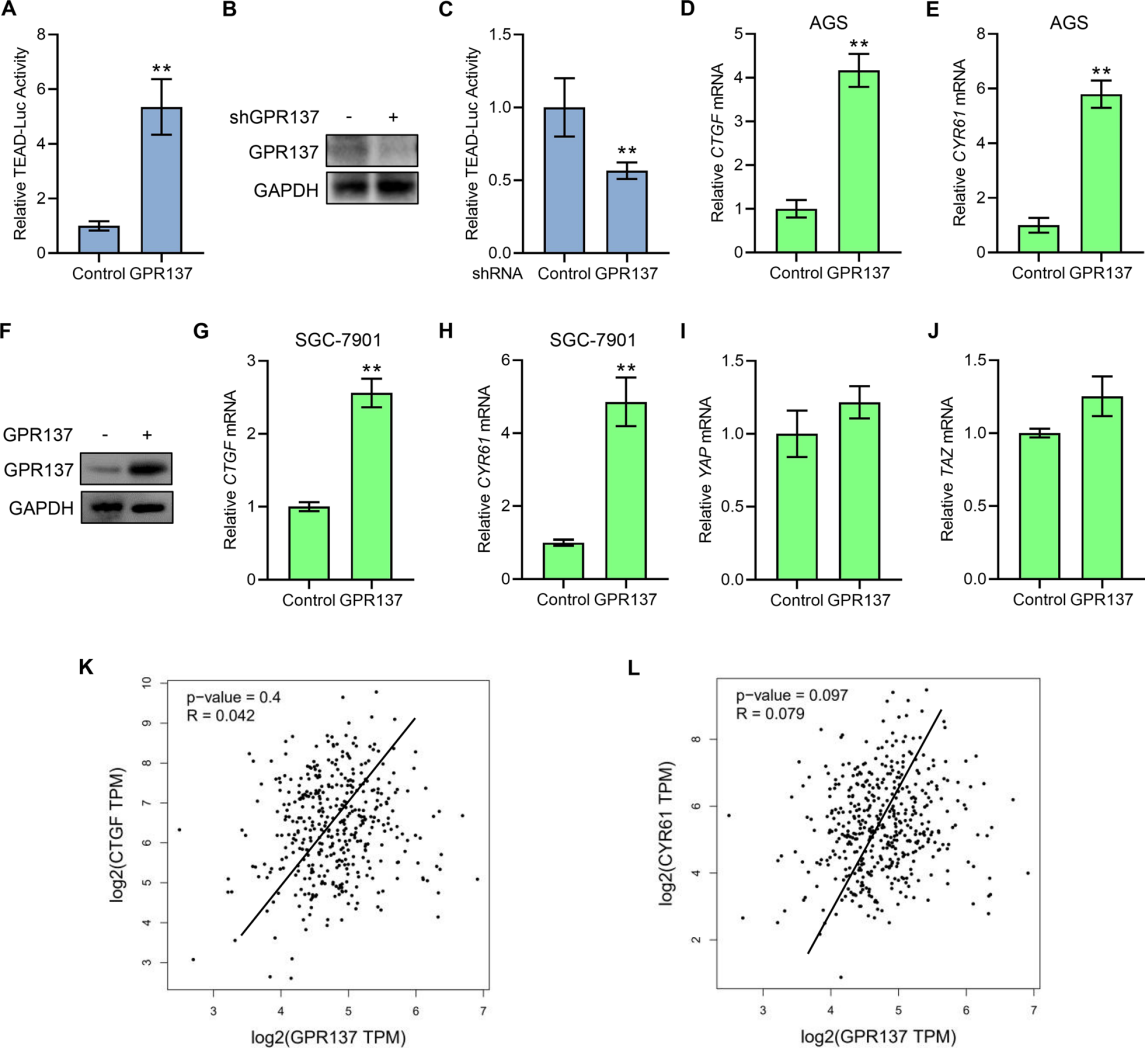
GPR137 inhibits Hippo signaling activity. **A** TEAD-luciferase assays in AGS cells transfected with GPR137 or an empty vector (control) for 24 h. **p < 0.01; error bar, SD. N = 3. **B** Protein levels of GPR137 in AGS cells transfected with GPR137 shRNA (shGPR137, +) or control scrambled shRNA (shGPR137, -) for 72 h. One representative of 3 independent blots is shown. **C** TEAD-luciferase assays in AGS cells transfected with GPR137 shRNA or control scrambled shRNA and culture for 72 h. **p < 0.01; error bar, SD. N = 3. **D**, **E** qRT-PCR assays for mRNA levels of *CTGF* (**D**) and *CYR61* (**E**) in AGS cells transfected with GPR137 or an empty vector (control) for 24 h. **p < 0.01; error bar, SD. N = 3. **F** Protein levels of GPR137 in SGC-7901 cells transfected with GPR137 (GPR137, +) or an empty vector (GPR137, -) for 24 h. One representative of 3 independent blots is shown. **G**, **H**) qRT-PCR assays for mRNA levels of *CTGF* **G** and *CYR61* (H) in SGC-7901 cells transfected with GPR137 or an empty vector (control) for 24 h. **p < 0.01; error bar, SD. N = 3. **I**, **J** qRT-PCR assays for mRNA levels of *YAP* (**I**) and *TAZ* (**J**) in AGS cells transfected with GPR137 or an empty vector (control) for 24 h. Error bar, SD. N = 3. **K**, **L** GEPIA database displayed the correlation between *GPR137* and *CTGF* (**K**) as well as *GPR137* and *CYR61* (**L**) in STAD

Hippo signaling target gene activation or inhibition can be measured by the TEAD-luciferase assays, whose readouts reflect the activity of Hippo signaling [24]. To next investigate the functional relationship between GPR137 and Hippo signaling, we conducted a reporter assay by ectopically co-expressing GPR137 vector and the 8xGT TEAD luciferase reporter plasmid in AGS cells, and found that GPR137 significantly stimulated the TEAD reporter activity (Fig. 4A). Conversely, knockdown of GPR137 by GPR137-shRNA that effectively suppressed the endogenous GPR137 protein levels markedly suppressed the TEAD-luciferase activity (Fig. 4B,C). Consistently, overexpression of GPR137 induced the mRNA expression of *CTGF* and *CYR61*, two bona fide TEAD target genes [25], in AGS cells (Fig. 4D,E), and the inductive effect of GPR137 on Hippo targets could be additionally verified in GPR137-transfected SGC-7901 cells (Fig. 4F-H). However, neither *YAP* mRNA expression nor *TAZ* mRNA expression was altered in the presence of GPR137 (Fig. 4I,J), indicating GPR137 may exert effects on Hippo activities through upstream levels, such as regulation of kinases, which controls YAP/TAZ activation and translocation but not transcription. Moreover, it is worth noting that, *GPR137* expression was positively correlated with *CTGF* expression as well as *CYR61* expression in STAD tissues (Fig. 4K,L), prompting us to further explore the regulation pattern of GPR137 and Hippo signaling in GC progression.

### GPR137 mediates Hippo activity through MST kinases

**Fig. 5:**
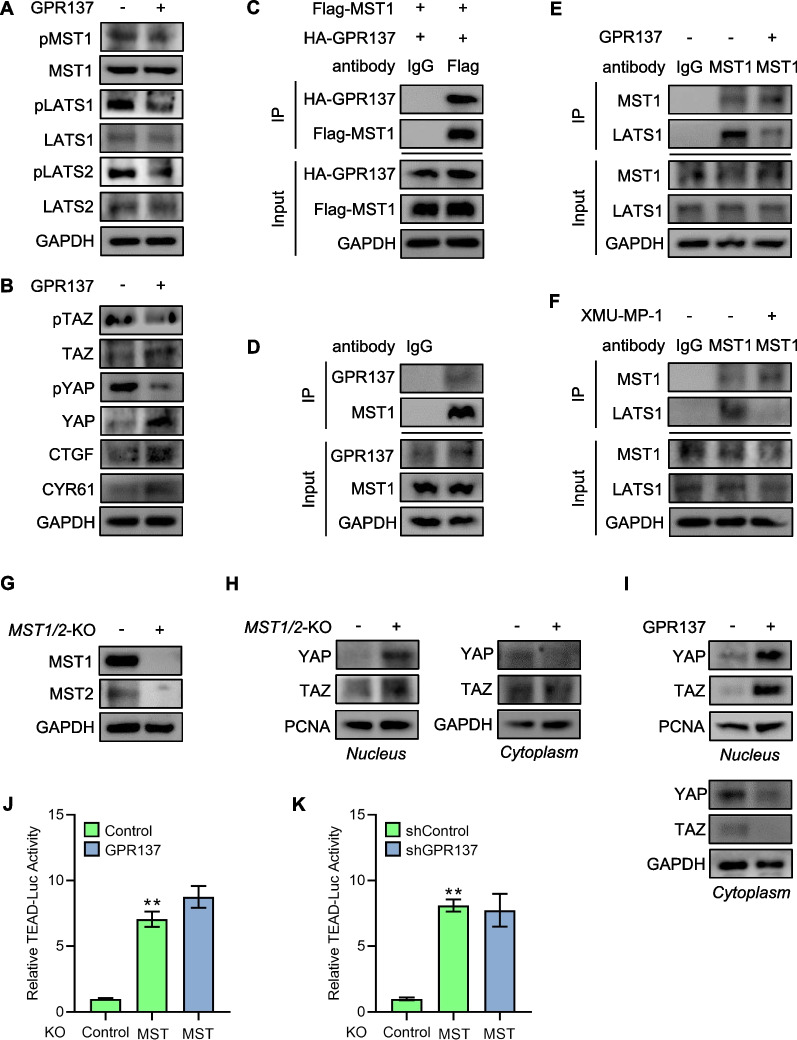
GPR137 suppresses Hippo signaling by MST. **A** Protein levels of pMST1, MST1, pLATS1, LATS1, pLATS2 and LATS2 in AGS cells transfected with GPR137 (GPR137, +) or an empty vector (GPR137, -) for 24 h. One representative of 3 independent blots is shown. **B** Protein levels of pTAZ, TAZ, pYAP, YAP, CTGF and CYR61 in AGS cells transfected with GPR137 (GPR137, +) or an empty vector (GPR137, -) for 24 h. One representative of 3 independent blots is shown. **C** Co-immunoprecipitation of HA-tagged GPR137 and Flag-tagged MST1 in HEK293T cells. IP: Flag; WB: HA. N = 3. **D** Co-immunoprecipitation of endogenous GPR137 and MST1 in AGS cells. IP: MST1; WB: GPR137. IgG was used as a negative control. One representative of 3 independent blots is shown. **E** Co-immunoprecipitation of endogenous MST1 and LATS1 in the presence of GPR137 in AGS cells. IP: MST1; WB: LATS1. IgG was used as a negative control. One representative of 3 independent blots is shown. **F** Co-immunoprecipitation of endogenous MST1 and LATS1 in the presence of XMU-MP-1 in AGS cells. IP: MST1; WB: LATS1. IgG was used as a negative control. One representative of 3 independent blots is shown. **G** Protein expression examination of MST1 and MST2 in *MST1/2*-knockout (*MST1/2*-KO) AGS cells or control AGS cells. One representative of 3 independent blots is shown. **H** Nucleo-cytoplasmic separation assays in *MST1/2*-knockout AGS (*MST1/2*-KO) cells or control cells, and protein levels of YAP and TAZ were detected. Proteins in nucleus (left) and in cytoplasm (right). One representative of 3 independent blots is shown. **I** Nucleo-cytoplasmic separation assays in GPR137-transfected AGS (GPR137, +) cells or control cells (GPR137, -), and protein levels of YAP and TAZ were detected. Proteins in nucleus (upper) and in cytoplasm (lower). One representative of 3 independent blots is shown. **J** TEAD-luciferase assays in *MST1/2*-knockout (*MST1/2*-KO) AGS cells or control AGS cells transfected with GPR137 or an empty vector (control). **p < 0.01; error bar, SD. N = 3. **K** TEAD-luciferase assays in *MST1/2*-knockout (*MST1/2*-KO) AGS cells or control AGS cells transfected with GPR137 shRNA or control scrambled shRNA. **p < 0.01; error bar, SD. N = 3

The up-regulated activity of Hippo signaling causes activation of MST1/MST2, which induces the phosphorylation and activation of LATS1/LATS2. Subsequently, LATS1/LATS2 phosphorylate YAP and TAZ, and YAP/TAZ phosphorylation by LATS1/LATS2 leads to their cytoplasmic sequestration and triggers further phosphorylation as well as ubiquitylation and proteasomal degradation, consequently suppressing the transcriptional output of the Hippo pathway [26]. In agreement with the results of TEAD-reporter assay (Fig. 4A,B) and regulation pattern of Hippo signaling, overexpression of GPR137 decreased the phosphorylation levels of MST1, LATS1 and LATS2, whereas the protein levels of MST1, LATS1 and LATS2 were not changed (Fig. 5A). In addition, overexpression of GPR137 down-regulated the phosphorylation levels of YAP and TAZ but up-regulated the protein levels of YAP and TAZ as well as the two targets, CTGF and CYR61 (Fig. 5B), demonstrating GPR137 negatively modulates Hippo pathway. Given that the MST core kinase functions up-stream of Hippo signaling [27], we thereby hypothesized that GPR137 might mediate MST. To this end, we tested for physical interactions between GPR137 and MST by immunoprecipitation. As expected, an interaction was observed by immunoprecipitation of Flag-MST1 and immunoblotting for HA-GPR137 in HEK293T cells (Fig. 5C). Similarly, an interaction between endogenous MST1 and GPR137 in AGS cells was also detected (Fig. 5D). Hippo signaling activation triggers MST kinase phosphorylation, which further binds with and phosphorylates the downstream kinase LATS [28], and, as an antagonist, XMU-XP-1 can suppress the MST1/2 signaling cascade by the disassociation of LATS from its binding with MST [29]. Intriguingly, we found that the interaction between LATS and MST was effectively reduced in the presence of GPR137 (Fig. 5E), which was comparable with the effect of XMU-XP-1 (Fig. 5F). To determine the requirement for MST kinases in GPR137-mediated Hippo inactivation, we thereby constructed AGS cells where both *MST1* and *MST2* genes were knocked out (*MST1/2*-KO) by CRISPR/Cas9 gene editing method (Fig. 5G). In agreement with a previous report [30], deficiency of *MST1* and *MST2* resulted in the accumulated YAP and TAZ protein in the nucleus (Fig. 5H). Similarly, overexpression of GPR137 gave rise to the obvious increase in nuclear distribution and decrease in cytoplasmic expression of YAP and TAZ (Fig. 5I). As expected, loss of *MST1* and *MST2* robustly induced the TEAD reporter activities, however, it is noticeable that neither overexpression of an exogenous GPR137 plasmid nor suppression of GPR137 by GPR137-shRNA significantly altered the TEAD-luciferase activities in *MST1/2*-KO AGS cells (Fig. 5J,K). Thus, GPR137 mediates Hippo activity through MST kinase.

### Loss of *GPR137* restrains AGS cell biological actions and enhances Hippo activity

**Fig. 6:**
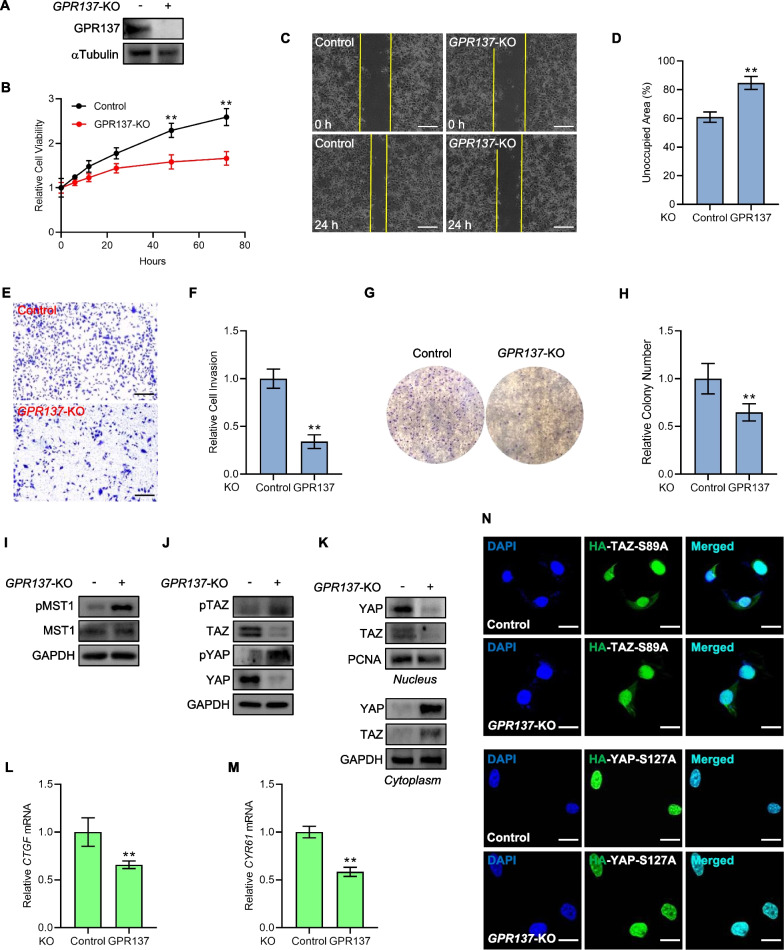
*GPR137* loss declines AGS cell malignancy but potentiates Hippo activity**. A** Protein expression examination of GPR137 in *GPR137*-knockout (*GPR137*-KO) AGS cells or control AGS cells. One representative of 3 independent blots is shown. **B** CCK-8 assays of GPR137-knockout AGS cells or control cells at different periods. **p < 0.01; error bar, SD. N = 3. **C** Wound healing assays of *GPR137*-knockout (*GPR137*-KO) AGS cells or control cells at 24 h. Bar = 100 μm. N = 3. **D** Statistical analysis of unoccupied area in (**C**). **p < 0.01; error bar, SD. **E** Matrigel invasion assays of *GPR137*-knockout (*GPR137*-KO) AGS cells or control cells at 24 h. Bar = 100 μm. N = 3. **F** Quantitative analysis of (**E**). **p < 0.01; error bar, SD. **G** Colony formation assays of *GPR137*-knockout (*GPR137*-KO) AGS cells or control cells. N = 3. **H** Quantitative analysis of (G). **p < 0.01; error bar, SD. **I** Protein levels of pMST1 and MST1 in *GPR137*-knockout (*GPR137*-KO) AGS cells or control cells. One representative of 3 independent blots is shown. **J** Protein levels of pTAZ, TAZ, pYAP and YAP in *GPR137*-knockout (*GPR137*-KO) AGS cells or control cells. One representative of 3 independent blots is shown. **K** Nucleo-cytoplasmic separation assays in *GPR137*-knockout AGS (*GPR137*-KO) cells or control cells, and protein levels of YAP and TAZ were detected. Proteins in nucleus (upper) and in cytoplasm (lower). One representative of 3 independent blots is shown. **L**, **M** qRT-PCR assays for mRNA levels of *CTGF* (L) and *CYR61* (M) in *GPR137*-knockout (*GPR137*-KO) AGS cells or control cells. **p < 0.01; error bar, SD. N = 3. **N** Immunofluorescent staining for HA-derived signal in *GPR137*-knockout (*GPR137*-KO) AGS cells or control cells transfected with HA-TAZ-S89A (upper) or HA-YAP-S127A (lower). Nuclei were stained with DAPI. Bar = 5 μm. N = 3

To further investigate the role of GPR137 in regulating the Hippo pathway in GC, we used CRISPR/Cas9 genome editing technology [11] to knockout *GPR137* in AGS gastric cancer cells. In contrast to the data of GPR137-overexpression (Fig. 2), knockout of *GPR137* (GPR137-KO) inhibited AGS cell growth at different time points (Fig. 6A,B), suppressed migration and invasion in AGS cells (Fig. 6C-F), and decreased AGS cell colony number (Fig. 6G,H). In accordance with TEAD-reporter data of GPR137 knockdown (Fig. 2B), knockout of *GPR137* exhibited the apparently up-regulated phosphorylation levels of MST1, YAP and TAZ and down-regulated protein levels of YAP and TAZ (Fig. 6I,J), which was in agreement with the regulation pattern of YAP/TAZ in Hippo signaling. In addition, in low-density cultured AGS cells, YAP and TAZ were mainly distributed in nucleus (Fig. 6K), however, *GPR137* deficiency gave rise to a marked decrease in nuclear YAP/TAZ and a dramatic increase in cytoplasmic expression of YAP/TAZ, which was assessed by a nucleo-cytoplasmic separation assay (Fig. 6K). As expected, the alterations in YAP/TAZ sub-cellular distribution were correlated with the down-regulated mRNA expression of the target genes, *CTGF* and *CYR61* (Fig. 6L,M). Of note, *GPR137* loss failed to affect the nucleo-cytoplasmic localization of either an HA-tagged gain-of-function mutant of TAZ (HA-TAZ-S89A) or an HA-tagged gain-of-function mutant of YAP (HA-YAP-S127A)(Fig. 6N), further proving that the effect of GPR137 on YAP/TAZ-mediated target genes is dependent on upstream kinases.

### GPR137 through Hippo signaling mediates AGS cell malignancy

**Fig. 7:**
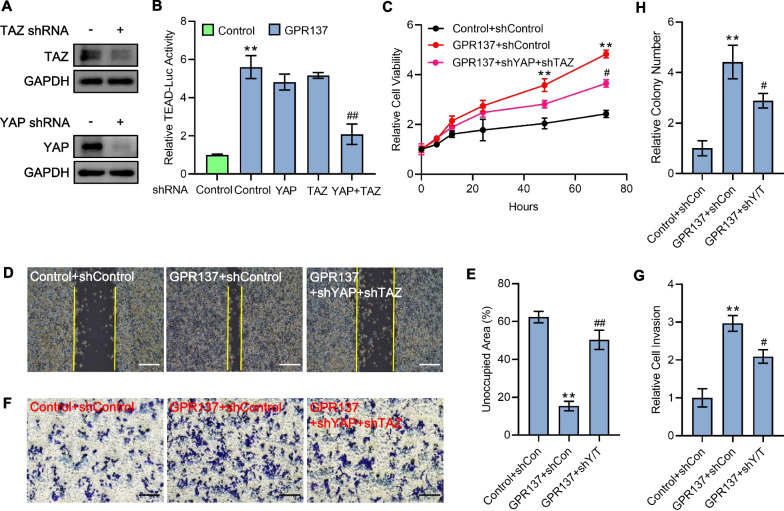
GPR137 elevates AGS cell malignancy through Hippo signaling. **A** Protein expression examination of YAP (upper) and TAZ (lower) in YAP-shRNA or TAZ-shRNA transfected AGS cells. One representative of 3 independent blots is shown. **B** TEAD-luciferase assays in AGS cells transfected with GPR137 or an empty vector (control) in combination with YAP shRNA, TAZ shRNA, or YAP shRNA and TAZ shRNA for 72 h. **, ^##^, *p* < 0.01; error bar, SD. N = 3. **C** CCK-8 assays in AGS cells transfected with GPR137 or an empty vector (control) in combination with YAP shRNA and TAZ shRNA at different periods. **p < 0.01; ^#^*p* < 0.05; error bar, SD. N = 3. **D** Wound healing assays in AGS cells transfected with GPR137 or an empty vector (control) in combination with YAP shRNA and TAZ shRNA (GPR137 + shY/T). N = 3. **E** Quantitative analysis of (**D**). **, ^##^, *p* < 0.01; error bar, SD. **F** Matrigel invasion assays in AGS cells transfected with GPR137 or an empty vector (control) in combination with YAP shRNA and TAZ shRNA (GPR137 + shY/T). N = 3. **G** Quantitative analysis of (**F**). **p < 0.01; ^#^*p* < 0.05; error bar, SD. (H) Quantitative analysis for colony formation assays in AGS cells transfected with GPR137 or an empty vector (control) in combination with YAP shRNA and TAZ shRNA (GPR137 + shY/T). **p < 0.01; ^#^*p* < 0.05; error bar, SD. N = 3

To figure out whether GPR137-mediated AGS cell malignancy is dependent on Hippo pathway, we constructed plasmids carrying shRNA sequences targeting YAP and TAZ, which markedly suppressed endogenous YAP and TAZ in AGS cells (Fig. 7A). Although knockdown of YAP or TAZ alone did not markedly alter the TEAD-luciferase activities (Fig. 7B), simultaneous abrogation of expression of YAP and TAZ not only significantly reversed the GPR137-induced TEAD-luciferase activities (Fig. 7B), but also effectively attenuated the GPR137-stimulated AGS cell proliferation (Fig. 7C) and capabilities of migration, invasion and colony formation (Fig. 7D–H), suggesting Hippo signaling participates in GPR137-regulated GC progression.

## Discussion

In this work, we show that the orphan receptor, GPR137, potentiates the GC cell malignancy through inactivation of Hippo signaling by forming a physical complex with MST kinase. Our results therefore identify the critical role of GPR137 in regulating GC progression.

Biomarkers for cancer prognosis could optimize treatment decisions and clinical outcomes for patients, and a number of studies have focused on searching for novel molecular prognostic biomarkers for GC. Among those, GPCRs have been reported to participate in cell proliferation, survival and motility, and their functions in tumor growth, angiogenesis, and metastasis also attract increasing attentions [31]. As an orphan GPCR-encoding gene that is expressed ubiquitously, *GPR137* is discovered by searching the Genbank genomic database [32]. Previous studies have identified that GPR137 is involved in the proliferation of tumor cells in several cancers, including ovarian [8], pancreatic [9], hepatoma [33], bladder [31], and prostate cancers [6], as well as medulloblastoma [34]. In addition to those tumors, our current study not only observes the up-regulated expression of GPR137 in STAD using online database, but also clarifies the function of GPR137 in regulating GC cell biological actions, including proliferation, migration, invasion, colony formation in vitro as well as xenograft growth in nude mice in vivo, suggesting that GPR137 could also be a potential therapeutic target for GC. To further figure out the way that GPR137 potentiates GC cell malignancy, RNA-sequencing was applied in our study. As a result, we focused on the down-stream effector, Hippo pathway. The Hippo signaling pathway plays a pivotal role in regulating cell survival, proliferation, differentiation, and organ size [35], and dysregulation of Hippo signaling gives rise to tumorigenesis, such as liver cancer [36], breast cancer [37], gallbladder cancer [38] as well as lung cancer [39] and gastric cancer [40]. Particularly, recent studies have identified that, as oncoproteins, the transcriptional co-activators, YAP/TAZ, are able to enhance cell proliferation, promote cell transformation, and increase cancer cell stemness. For example, in breast cancer, YAP/TAZ can induce cancer cell proliferation and reduce cancer cell death, which congruously lead to the increased cancer cell numbers [41]. YAP/TAZ can also promote cancer cell transformation. A previous report has found that overexpression of YAP in human non-transformed mammary epithelial cells induces epithelial-to-mesenchymal transition (EMT), and consistently, the up-regulated TAZ expression in mammary cells results in the acquisition of a spindle-shaped morphology and the elevated capacity of cell migration and invasion [42]. Our current findings have further added the function of YAP/TAZ in GC cells, demonstrating that YAP/TAZ participates in GC development by regulating cancer cell proliferation, migration, and invasion. Thus, the enhanced YAP/TAZ activity/expression may promote cancer progression by multiple approaches, such as modulating cell proliferation and motility. Although investigation of Hippo regulation by GPR137 in our study was primarily based on the RNA-sequencing data in AGS cells, our data repeated in SGC-7901 cells clearly revealed the similar effect of Hippo signaling by GPR137 (Fig. 4G,H), reinforcing the notion that GPR137 contributes to GC progression by modulating Hippo signaling. In addition to our findings of GPR137 herein, whether other GPCRs participate in GC carcinogenesis by regulating Hippo needs to be further tested and determined.

As the important transcriptional co-activators, YAP and TAZ both can bind with TEAD and take part in regulating TEAD-mediated transcription of target genes [43]. Intriguingly, some previous studies have found that the one activator could also exert effects on the other. Finch-Edmondson et al. reported that TAZ protein accumulation is negatively regulated by YAP abundance in mammalian cells [44]. In agreement with those studies, in our study, inhibition of YAP or TAZ alone did not alter the GPR137-induced TEAD reporter activity, which was significantly restored by double knockdown simultaneously (Fig. 7A), suggesting that some compensatory mechanism may also exist between YAP and TAZ in GC cells. Thus, with the right strategy and impetus, it would be promising to develop novel drug to combat both YAP and TAZ in cancers in the near future.

The mammalian MST (Mammalian Sterile20-like) kinase family, which is related to the Hippo kinase in *Drosophila melanogaster*, acts as key the signaling molecule that mediates cell proliferation, cell migration and cell polarity in development and disease progression including cancer [30]. Given the effect of MST/LATS kinase signaling networks on cell proliferation and motility, these kinases have been found to play important roles in various cancers. In 2015 the exome sequencing has revealed some mutations and fusions of MST1/2 or LATS1/2 kinases in cancer, for example LATS1 fusion in mesothelioma [45]. In addition, as the up-stream regulator in Hippo signaling, the phosphorylation status of MST kinase has a profound impact on the consequent Hippo signaling activity. A previous report has indicated that the Sterile 20 family kinase, Tao-1, phosphorylates Hippo/MST kinases to regulate the Hippo-Salvador-Warts tumor suppressor pathway to restrict cell proliferation in developing imaginal epithelial [46]. In contrast, Heidary Arash et al*.* found that MARK4 inhibits Hippo signaling by regulating MST phosphorylation levels to promote proliferation and migration of breast cancer cells [47]. In this work, we have elucidated that GPR137 inhibits Hippo signaling by suppressing MST kinase phosphorylation and its binding to LATS in GC cells, providing with the evidence that MST kinase activity is closely associated with cancers. Nevertheless, further study is in need to figure out the binding pattern of MST by GPR137 (determination of direct or indirect interaction as well as binding domain or motif) and to confirm whether this molecular mechanism functions in other cancer cells, and it would be interesting to search for other important upstream regulators of the Hippo signaling pathway to understand how they link to the Hippo regulatory pathways.

## Conclusions

Overall, by human gastric cancer cell model, xenograft model and clinical tissue samples, the present study suggests that the GPR137-Hippo signaling plays a key role in regulating GC cell malignancy. Thus, our findings suggest that targeting GPR137 might provide therapeutic benefit in GC.

## Data Availability

All data generated or analyzed during this study are included in this published article.

## Abbreviations

CCK-8: Cell Counting Kit-8
DAB: Diaminobenzidine
DAPI: 4',6-Diamidino-2-phenylindole
DMEM: Dulbecco’s modified Eagle’s medium
EMT: Epithelial-mesenchymal transition
FBS: Fetal bovine serum
GC: Gastric cancer
GEPIA: Gene Expression Profiling Interactive Analysis
GO: Gene Ontology
GPCR: G protein-coupled receptor
GPR137: G protein-coupled receptor 137
HET293T: Human embryonic kidney 293T
KEGG: Kyoto Encyclopedia of Genes and Genomes
MST: Mammalian Sterile20-like
qRT-PCR: Quantitative reverse transcription-PCR;
STAD: Stomach adenocarcinoma
